# To B or Not to B: Comparative Genomics Suggests *Arsenophonus* as a Source of B Vitamins in Whiteflies

**DOI:** 10.3389/fmicb.2018.02254

**Published:** 2018-09-25

**Authors:** Diego Santos-Garcia, Ksenia Juravel, Shiri Freilich, Einat Zchori-Fein, Amparo Latorre, Andrés Moya, Shai Morin, Francisco J. Silva

**Affiliations:** ^1^Department of Entomology, The Hebrew University of Jerusalem, Rehovot, Israel; ^2^Institute of Plant Sciences, Newe-Ya'ar Research Center, Agricultural Research Organization, Ramat-Yishai, Israel; ^3^Department of Entomology, Newe-Ya'ar Research Center, Agricultural Research Organization, Volcani Center, Ramat-Yishai, Israel; ^4^Institute for Integrative Systems Biology, Universitat de València-CSIC, València, Spain; ^5^Unidad Mixta de Investigación en Genómica y Salud, Fundación para el Fomento de la Investigación Sanitaria y Biomédica de la Comunidad Valenciana (FISABIO) and Institute for Integrative Systems Biology, Universitat de València, València, Spain

**Keywords:** whitefly, symbiosis, vitamins, genome reduction, *Arsenophonus*, *Wolbachia*, metabolic complementation, riboflavin

## Abstract

Insect lineages feeding on nutritionally restricted diets such as phloem sap, xylem sap, or blood, were able to diversify by acquiring bacterial species that complement lacking nutrients. These bacteria, considered obligate/primary endosymbionts, share a long evolutionary history with their hosts. In some cases, however, these endosymbionts are not able to fulfill all of their host's nutritional requirements, driving the acquisition of additional symbiotic species. Phloem-feeding members of the insect family Aleyrodidae (whiteflies) established an obligate relationship with *Candidatus* Portiera aleyrodidarum, which provides its hots with essential amino acids and carotenoids. In addition, many whitefly species harbor additional endosymbionts which may potentially further supplement their host's diet. To test this hypothesis, genomes of several endosymbionts of the whiteflies *Aleurodicus dispersus, Aleurodicus floccissimus* and *Trialeurodes vaporariorum* were analyzed. In addition to *Portiera*, all three species were found to harbor one *Arsenophonus* and one *Wolbachia* endosymbiont. A comparative analysis of *Arsenophonus* genomes revealed that although all three are capable of synthesizing B vitamins and cofactors, such as pyridoxal, riboflavin, or folate, their genomes and phylogenetic relationship vary greatly. *Arsenophonus* of *A. floccissimus* and *T. vaporariorum* belong to the same clade, and display characteristics of facultative endosymbionts, such as large genomes (3 Mb) with thousands of genes and pseudogenes, intermediate GC content, and mobile genetic elements. In contrast, *Arsenophonus* of *A. dispersus* belongs to a different lineage and displays the characteristics of a primary endosymbiont—a reduced genome (670 kb) with ~400 genes, 32% GC content, and no mobile genetic elements. However, the presence of 274 pseudogenes suggests that this symbiotic association is more recent than other reported primary endosymbionts of hemipterans. The gene repertoire of *Arsenophonus* of *A. dispersus* is completely integrated in the symbiotic consortia, and the biosynthesis of most vitamins occurs in shared pathways with its host. In addition, *Wolbachia* endosymbionts have also retained the ability to produce riboflavin, flavin adenine dinucleotide, and folate, and may make a nutritional contribution. Taken together, our results show that *Arsenophonus* hold a pivotal place in whitefly nutrition by their ability to produce B vitamins.

## 1. Introduction

Mutualistic, commensal, or parasitic relationships have been described in a diverse array of eukaryotes that are allied with bacterial symbionts (Moya et al., 2008). The class Insecta provides examples of mutualistic associations, and several lineages are known to have lived in intimate relationships with obligate symbionts for millions of years. Such symbionts are usually harbored inside specialized cells, termed bacteriocytes (sometimes organized as a bacteriome), and are vertically transmitted from the mother to her offspring. These bacterial symbionts, living inside cells and transmitted throughout long evolutionary periods, have been denoted primary (or obligatory) endosymbionts. The evolution of these symbioses has had several effects on the gene repertoires of both hosts and endosymbionts, but is most often characterized by drastic reductions in the genome contents of the latter (Latorre and Manzano-Marín, 2016). Host diet complementation frequently initiates the symbiosis, but in some cases, the host has nutritional requirements which the primary endosymbiont is unable to satisfy, and secondary (or facultative) endosymbionts are required for further diet complementation. When these requirements become vital, a secondary endosymbiont might become primary, resulting in a consortium of primary endosymbionts (Lamelas et al., 2011).

Aphids (Aphidoidea), scale insects (Coccoidea), whiteflies (Aleyrodoidea), and psyllids (Psylloidea) are superfamilies composing the suborder Sternorrhyncha, included in the order Hemiptera. All Sternorrhyncha feed on plant phloem sap, a diet poor in nitrogenous compounds, especially essential amino acids, but rich in sugars (Douglas, 2006). Among them, whiteflies and psyllids are two sister lineages whose common ancestor established a long-term association with a bacterium of the family Halomonadaceae (Gammaproteobacteria:Oceanospirillales) (Santos-Garcia et al., 2014a). This bacterial lineage has co-diversified with its hosts and is known as *Candidatus* Portiera aleyrodidarum in whiteflies and *Candidatus* Carsonella ruddii in psyllids (hereafter, the term *Candidatus* is used only the first time a species is mentioned). These two endosymbionts have extremely reduced genomes, ranging from 160 to 174 kb and 182–207 genes in *Carsonella*, and 280–357 kb and 284–318 genes in *Portiera* (Sloan and Moran, 2012b; Santos-Garcia et al., 2015). Based on gene repertoires of both *Carsonella* and its host, the two partners can complement each other to produce all essential amino acids, with the exception of tryptophan and histidine (Sloan et al., 2014). Indeed, tryptophan complementation by *Carsonella* and a secondary endosymbiont has been only reported in a few psyllid species (Sloan and Moran, 2012b). On the other hand, it was proposed that *Portiera* collaborates with its hosts in the synthesis of all essential amino acids, and is able to produce carotenoids and lipoate independently (Santos-Garcia et al., 2012, 2015; Sloan and Moran, 2012a; Luan et al., 2015). Neither *Carsonella* and nor *Portiera* can produce other vitamins or cofactors, which are deficient in the phloem sieve-element diet of whiteflies and psyllids (Zimmermann and Milburn, 1975). Unlike the generally limited bacterial associates of psyllids, individual whiteflies may carry one or several additional endosymbionts belonging to seven genera: the gammaproteobacteria *Hamiltonella* (Enterobacterales:Enterobacteriaceae) and *Arsenophonus* (Enterobacterales:Morganellaceae); the alphaproteobacteria *Wolbachia* (Rickettsiales:Anaplasmataceae), *Rickettsia* (Rickettsiales:Rickettsiaceae), and *Hemipteriphilus* (Rickettsiales:Rickettsiaceae); the bacteroidetes *Cardinium* (Cytophagales:Amoebophilaceae); and the chlamydiae *Fritschea* (Parachlamydiales: Simkaniaceae) (Thao et al., 2003; Bing et al., 2013b; Skaljac et al., 2013; Marubayashi et al., 2014; Zchori-Fein et al., 2014). It has been suggested that some of these additional endosymbionts are involved in supplying vitamins and cofactors to their host. For example, *Candidatus* Hamiltonella defensa from the whitefly *Bemisia tabaci* potentially produces several B vitamins (riboflavin, pyridoxine, biotin and folic acid) (Rao et al., 2015). *Hamiltonella* is fixed in the MEAM1 and MED-Q1 species of the *B. tabaci* complex and is always found sharing bacteriocytes with *Portiera* (Gottlieb et al., 2008; Zchori-Fein et al., 2014). Interestingly, *Arsenophonus* is a genus of secondary endosymbiont that has been observed in many whitefly species (Thao and Baumann, 2004; Cass et al., 2014; Marubayashi et al., 2014; Zchori-Fein et al., 2014; Pandey and Rajagopal, 2015; Santos-Garcia et al., 2015). Similar to *Hamiltonella, Arsenophonus* is restricted to the bacteriocytes and seems to be fixed in the whitefly species/population. In contrast, other endosymbionts such as *Candidatus* Wolbachia or *Candidatus* Rickettsia species present different tissue tropisms and are generally not fixed (Gottlieb et al., 2008; Skaljac et al., 2010, 2013; Kapantaidaki et al., 2014; Marubayashi et al., 2014; Zchori-Fein et al., 2014). Despite some potential complementations with *Portiera* for essential amino acid production detected in *Rickettsia*, the production of threonine in *Wolbachia*, or a few intermediate metabolites and cofactors in both endosymbionts, *Wolbachia* and *Rickettsia* from *B. tabaci* have limited metabolic potential and seem to import more resources from the host than they can share with it (Opatovsky et al., 2018). Similarly, *Cardinium* from *B. tabaci* demonstrates scarce metabolic potential and seems not to be involved in host diet complementation (Santos-Garcia et al., 2014b). On the other hand, some *Arsenophonus* and *Wolbachia* lineages have evolved intimate associations with other insect taxa with different diets, such as blood-sucking insects, where the supplementation of B vitamins and cofactors are their proposed role (Hosokawa et al., 2010; Šochová et al., 2017).

The sole whitefly family, Aleyrodidae, is comprised of two main subfamilies – the Aleurodicinae and the Aleyrodinae. These two subfamilies probably originated between the Jurassic (Shcherbakov, 2000; Drohojowska and Szwedo, 2015) and Middle Cretaceous (Campbell et al., 1994). Whereas the Aleyrodinae contains the largest number of species described to date (140 genera, 1440 species), the Aleurodicinae has a relatively low number of described species (17 genera, 120 species) (Ouvrard and Martin, 2018). Analysis of the genome content of *Portiera* from two Aleurodicinae species, *Aleurodicus dispersus* and *Aleurodicus floccissimus*, and two Aleyrodinae species, *Trialeurodes vaporariorum* and *B. tabaci*, suggested that they supply their hosts with essential amino acids and carotenoids but are unable to complement their hosts' diet with essential vitamins and cofactors (Santos-Garcia et al., 2015). Because *Hamiltonella* potentially supplies *B. tabaci* with vitamins and cofactors (Rao et al., 2015), the work presented here was initiated to test the prediction that *Arsenophonus* and/or *Wolbachia*, are capable of providing their hosts with the lacking components.

## 2. Materials and methods

### 2.1. Sequence retrieval

Sequences of bacteria other than *Portiera* were retrieved from a previous shotgun sequencing project of three whitefly species: *A. dispersus, A. floccissimus*, and *T. vaporariorum* (Santos-Garcia et al., 2015). Briefly, *T. vaporariorum* was collected in 2014 near the IRTA Institute of Agrifood Research and Technology (Barcelona, Spain) and identified by Dr. Francisco José Beitia. *A. dispersus* and *A. floccissimus* were collected and identified by Dr. Estrella Hernandez Suarez in 2014 from banana fields (Tenerife Island, Spain). All three whiteflies harbored *Portiera, Arsenophonus* and *Wolbachia* endosymbionts. Genomic DNA (gDNA) was obtained using an alkaline lysis method from single bacteriomes (dissected with glass microneedles). For each species, 10 single bacteriome gDNA extractions were subjected to whole-genome amplification (GenomiPhi V2, GE Healthcare) to representatively increase the total amount of gDNA (from both host and endosymbionts) before sequencing, and pooled by species. Whole-genome amplified gDNA was sequenced by Illumina HiSeq 2000 using a mate-paired library (100 bpX2, 3 kb insert size). A full description can be found in Santos-Garcia et al. (2015).

### 2.2. Metagenome-assembled genomes

For each species library, RAW Illumina reads were quality checked, and trimmed/clipped if necessary, with FastQC v0.11.3 (Andrews, 2010) and TrimmomaticPE v0.33 (Bolger et al., 2014), respectively. Kraken v0.10.6 (Wood and Salzberg, 2014) was used to classify the RAW Illumina reads with a custom genomic database including: *Portiera, Hamiltonella, Rickettsia, Wolbachia, Arsenophonus, Candidatus* Cardinium hertigii, several whiteflies' mitochondria, *Bemisia tabaci* MEAM1, and *Acyrthosiphon pisum* (Table S1). Reads classified as mitochondrial, insect or *Portiera* were discarded. The remaining reads were assembled with SPADES v3.11.0 (–meta –careful –mp) (Nurk et al., 2017). The resultant contigs were classified using Kraken and the custom database. Contigs belonging to known whitefly endosymbionts were recovered and added to the custom database. Trimmed/clipped reads were reclassified and reads belonging to known whitefly endosymbionts were reassembled alone with SPADES (–careful –mp) to produce metagenome-assembled genomes (MAGs). Illumina reads were digitally normalized (khmer v1.1) before the reassembly stage (Crusoe et al., 2015). MAG contigs were scaffolded and gap-filled with SSPACE v3 (Boetzer et al., 2011) and Gapfiller v1.10 (Boetzer and Pirovano, 2012), respectively. Finally, a manual iterative mapping approach, using Illumina trimmed/clipped reads, was performed with Bowtie2 v2.2.6 (Langmead and Salzberg, 2012), MIRA v4.9.5 (mapping mode) (Chevreux et al., 1999), and Gap4 (Staden et al., 2000) until no more contigs/reads were joined/recovered for each MAG. Finally, Bowtie2 and Pilon v.1.21 (–jumps) (Walker et al., 2014) with Illumina reads were used to correct MAG contigs.

### 2.3. MAG annotation

Initial annotations of MAGs were performed with prokka v1.12 (Seemann, 2014). Enzyme commission numbers were added with PRIAM March-2015 release (Claudel-Renard et al., 2003). Gene Ontology, PFAM, and InterPro terms were added with InterProScan v5.27-66 (Jones et al., 2014). Putative pseudogenes and their positions in the genome were detected with LAST using several bacterium-related proteomes as query (Kiełbasa et al., 2011). Insertion sequences were predicted with ISsaga (Varani et al., 2011). *B. tabaci* genome annotations (GCA_001854935.1) were downloaded and refined with PRIAM and InterProScan. MAGs, and their corresponding *Portiera* (Santos-Garcia et al., 2015) and *B. tabaci* metabolism comparisons were performed on PathwayTools v21.5 (Karp et al., 2002). Pathway completeness was measured as the number of enzymes present in the analyzed genome over the total number of enzymes in the pathway. Heat map and clustering were performed on R (R Core Team, 2018) with ggplots2 (Wickham, 2009).

### 2.4. Comparative genomics

OrthoMCL v2.0.9 was used to compute clusters of orthologous proteins (Li et al., 2003). Cluster of orthologous groups (COG) terms were assigned using DIAMOND v0.8.11.73 (July 2016 bacterial RefSeq database, Buchfink et al., 2015) and MEGAN6 (Huson et al., 2016). Average nucleotide identity (ANI) and average amino acid identity (AAI) values were obtained with the Enveomics tools (Rodriguez-R and Konstantinidis, 2016). Synteny between MAGs was checked with Mummer v3 (Kurtz et al., 2004) and Mauve, using *Arsenophonus* from *A. dispersus* (ARAD) or *Wolbachia* from *A. dispersus* (WBAD) as the reference for contig re-ordering (Darling et al., 2010). GenoPlotR was used to plot the Mauve results (Guy et al., 2010).

The presence of potential orthologs of *B. tabaci* genes in the other whitefly species was assessed using the Illumina cleaned data and DIAMOND, with a blastx search strategy (minimum alignment length of 25 bp and 1e^−3^* e*-value) against the selected *B. tabaci* proteins (Table S2). Recovered reads were classified with Kraken, using the custom and mini-Kraken databases, to discard bacterial reads. Finally, non-bacterial reads were used as query for a blastx search against the nr database (last access: March 2018) (Altschul et al., 1990) and their best hit was classified with MEGAN6. Only reads assigned to the phylum Arthropoda were considered to be genomic reads of the screened genes in the other whitefly species.

### 2.5. Phylogenetics

General phylogenetic position of the newly sequenced *Arsenophonus* and *Wolbachia* were assessed using several 16S rRNA genes downloaded from GenBank. Genes were aligned with ssu-align (default masking) (Nawrocki, 2009). IQ-TREE v1.6.2 was used to select the best substitution model (SYM+R3 and TN+F+R2, respectively) and to infer the majority rule maximum likelihood (ML) tree, with its associated support values (Nguyen et al., 2015; Kalyaanamoorthy et al., 2017).

Phylogenomic trees were generated using 82 and 87 conserved proteins, selected using PhyloPhlAn v0.99 (Segata et al., 2013), from *Arsenophonus* and *Wolbachia* inferred proteomes, respectively. Protein files were sorted by species name, using fastasort from exonerate v2.2.0 (Slater and Birney, 2005) and aligned with MAFFT v7.215 (Katoh et al., 2002). Protein alignments were concatenated by species index using Geneious v11.1.2 (Kearse et al., 2012). IQ-TREE was used to infer the majority rule ML tree, and associated support values, under the substitution models JTTDCMut+F+R3 for *Arsenophonus* and JTT+F+I+G4 for *Wolbachia*.

## 3. Results

### 3.1. General genomic features

**Figure 1:**
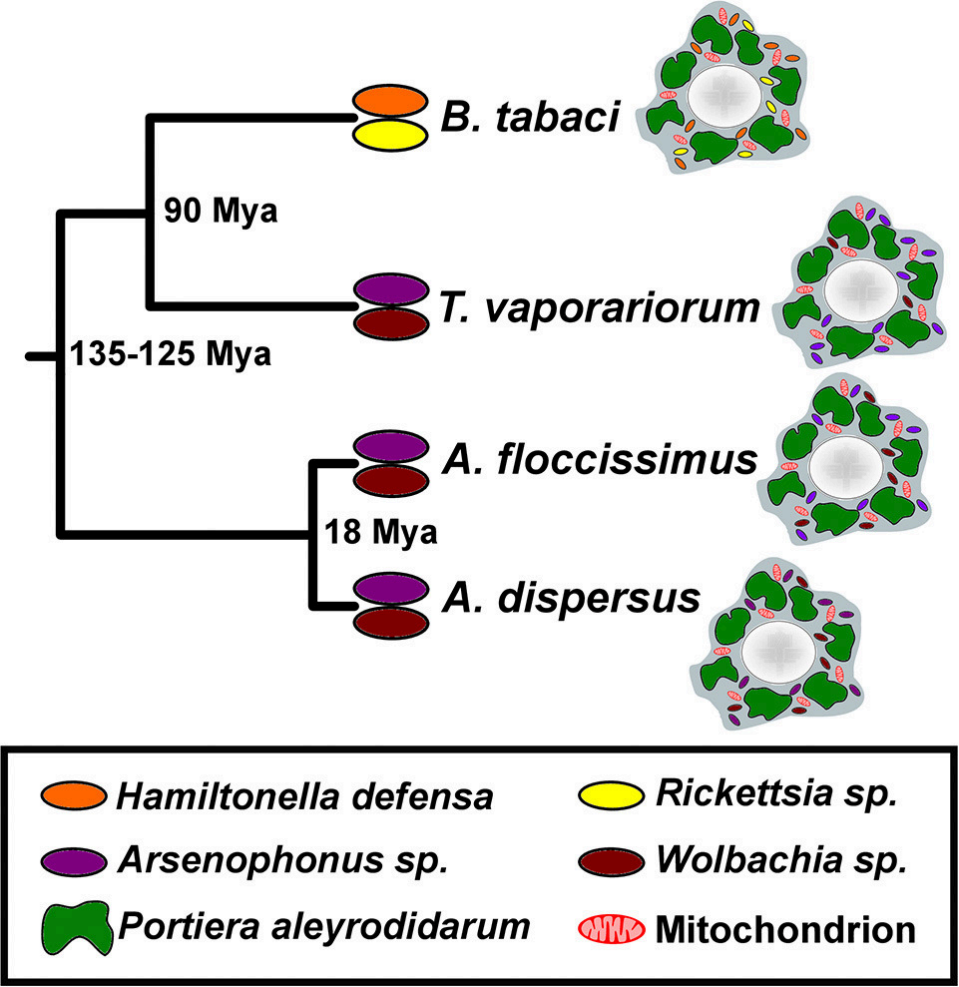
Whiteflies and endosymbiont content. Schematic representation of the phylogeny and endosymbionts content of the *Aleurodicus, T. vaporariorum* and *B. tabaci* whiteflies (Santos-Garcia et al., 2015). Sequences from *Wolbachia* were scarcely detected in *T. vaporariorum*, suggesting low amounts of this bacterium in the insect. Mya, million years ago.

A total of five endosymbiont MAGs were recovered from the three studied whitefly species: *Arsenophonus* from *A. dispersus* (ARAD), *A. floccissimus* (ARAF) and *T. vaporariorum* (ARTV), and *Wolbachia* from *A. dispersus* (WBAD) and *A. floccissimus* (WBAF) (Figure 1 and Table 1). All MAGs were of draft status but with different assemblage quality. Only ARAD was assembled as a circular scaffold, supported by some mate-paired reads. However, a gap at the contig edges was not closed due to the presence of a *dnaX* duplication and a long-AT low-complexity region. The assembly of the ARAF genome was also of high quality, with only 11 contigs and a N50 value ≥500 kb. The rest of the recovered MAGs were of different draft statuses, with *Wolbachia* being the most fragmented. Although *Wolbachia* had been previously detected by PCR in *T. vaporariorum* (Santos-Garcia et al., 2015), its genome was impossible to assemble and analyze due to the low amount of recovered reads (Table S3). Indeed, only 21 *Wolbachia* contigs were recovered. Of these, only one contig was ≥1 kb (2.5X coverage) while the rest were ≤400 nt. This suggests that *Wolbachia* is present in *T. vaporariorum* but in low amounts. However, an alternative explanation is that recovered *Wolbachia* reads/contigs in *T. vaporariorum* resulted from sequence index misassignments during multiplex sequencing (index switching).

**Table 1:** Assembly statistics and genomic features of the five endosymbionts sequenced.

	**ARAD**	**ARAF**	**ARTV**	**WBAD**	**WBAF**
Species	*Arsenophonus* sp.	*Arsenophonus* sp.	*Arsenophonus* sp.	*Wolbachia* sp.	*Wolbachia* sp.
Host	*A. dispersus*	*A. floccissimus*	*T. vaporariorum*	*A. dispersus*	*A. floccissimus*
Genome size (bp)	663,125	3,001,875	3,080,136	1,244,696	1,239,495
Contigs	1	11	93	237	422
N50 (bp)	-	510,728	236,293	9,433	5,991
Coverage	1,698	2,500	182	268	106
%GC	32	37	37	34	34
Genes	428	1,880	2,312	990	909
CDS	381	1,781	2,209	946	862
Noncoding RNA genes	47	99	103	44	47
Coding density^†^	54	41	51	70	58
Avg. CDS length	941	689	712	918	840
CDS %GC	36	39	39	34	34
1st+2nd %GC	42	42	42	38	38
3rd %GC	23	31	32	26	26
Pseudogenes	274	1,213	946	346	638
Avg. pseudo length	461	1,030	1,011	586	527
Pseudo %GC	30	38	38	34	35
rRNA (16S|23S|5S)	1|1|3	1|1|4	1|0|9^*^	1|1|1	1|1|1
tRNA	34	43	43	34	32
tmRNA	1	1	1	1	1
other RNAs	7	49	49	6	11
Insertion Sequences (IS)	0	11	21^*^	5^*^	2^*^
Prophage regions	0	12	12	2	0
Prophage regions size (bp)	-	430,067	336,650	9,948	-
Complete Prophage	-	7	7	0	-
Incomplete Prophage	-	5	5	2	-

* rRNA genes and ISs could be underestimated due to the draft status of the assembly

† Total number of nucleotides in functional CDS/total number of nucleotides in the genome

According to their genomic features, the sequenced *Arsenophonus* could be divided into two groups: ARAD displayed the typical characteristics of primary endosymbionts, whereas ARAF and ARTV displayed genomic features closer to facultative or secondary endosymbionts (Table 1). This pattern was emphasized by the different genome sizes (0.67 vs. 3 Mb for ARAD vs. ARAF and ARTV), and their respective GC content (32 vs. 37%), numbers of genes (428 vs. around 2000), and presence of insertion sequences and prophages (0 vs. 12). However, both groups had a low coding density (range 41–54%) due to the presence of almost as many pseudogenes as genes. Most of the prophages in ARAF and ARTV were unrelated (based on their ANI values) except the pairs of prophages 2/8 and 3/7 from ARAF and the pair of prophage 9 from ARAF and prophage 7 from ARTV (Figures S1, S2 and Table S4).

Regarding *Wolbachia*, both recovered MAGs displayed sizes and genomic features similar to other sequenced species from this genus (Ellegaard et al., 2013), with WBAF being more fragmented and lacking two tRNAs. Two incomplete prophages were detected in the genome of WBAD (Figure S1).

### 3.2. Phylogenetic placement

**Figure 2:**
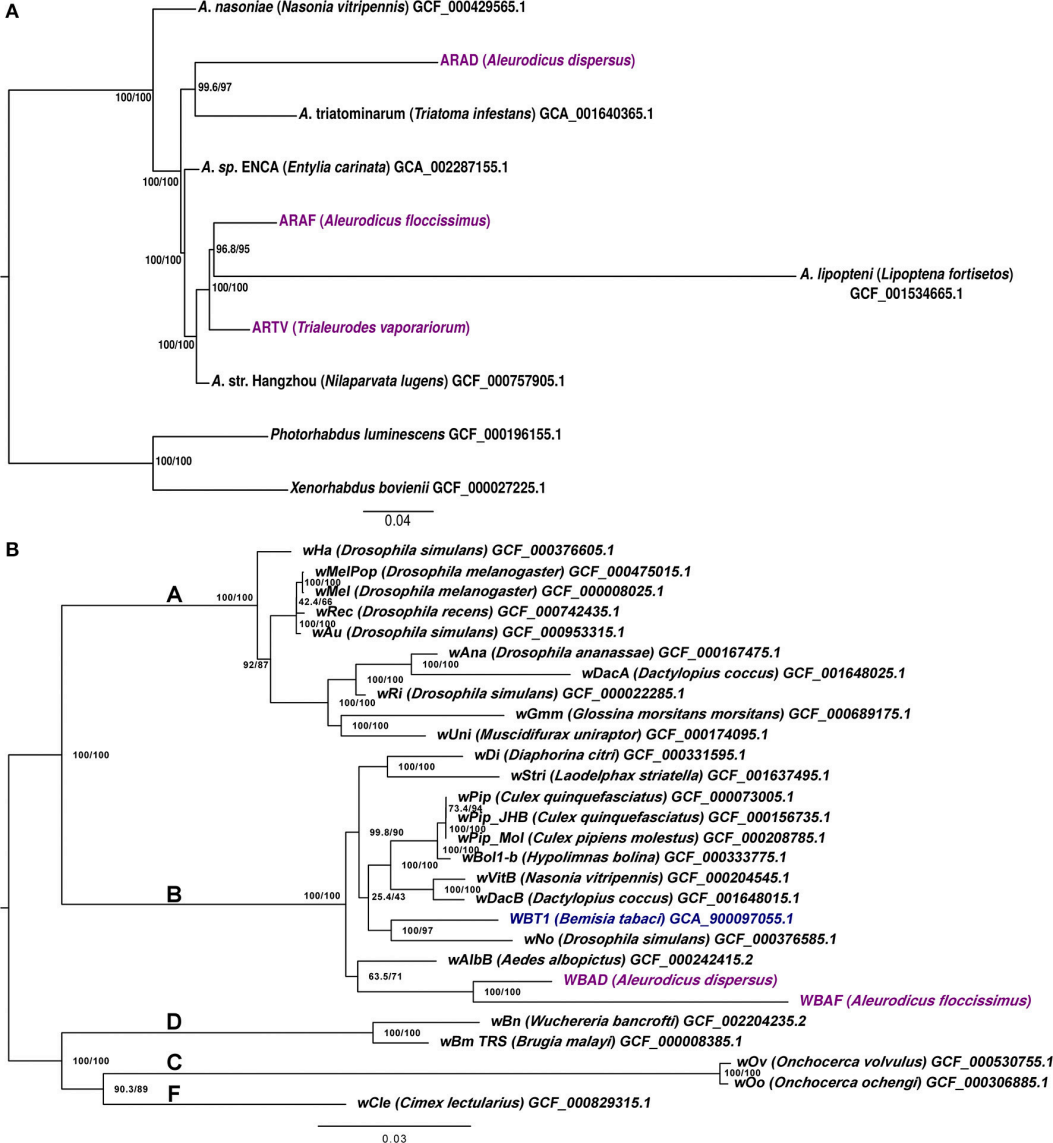
Phylogenomic trees of *Arsenophonus* and *Wolbachia*. **(A)*** Arsenophonus* (rooted) and **(B)*** Wolbachia* (unrooted) ML trees were inferred using 82 and 87 concatenated conserved protein alignments under a JTTDCMut+F+R3 and JTT+F+R3 substitution model, respectively. Support values were obtained with 5000 ultrafast bootstraps (right node labels) and 5000 SH-aLRT (left node labels). Names for the eukaryotic hosts are shown after the strain names in parentheses. Accession numbers are displayed for each of the proteomes used. *Arsenophonus* ARAD, ARAF, and ARTV and *Wolbachia* WBAD and WBAF are highlighted in purple. **(B)** Bold face letters denote the different *Wolbachia* supergroups. *Wolbachia* from *Bemisia tabaci* is highlighted in blue.

The phylogenetic positions of each assembled *Arsenophonus* within the genus were tested using 16S rRNA phylogeny, phylogenomics and ANI/AAI analyses. Based on the 16S rRNA phylogenetic analysis, *Arsenophonus* from whiteflies were distributed in at least five supported clusters, with ARAD being in a different lineage than ARAF and ARTV (Figure S3). ARAD was placed in a cluster together with other *Arsenophonus* from the whiteflies *A. dispersus, Aleurodicus dugesii*, and *Bemisia centroamericana*, and *Candidatus* Arsenophonus triatominarum from several triatomine bugs, among other insects. ARAF and ARTV were close to an *Arsenophonus* from *T. vaporariorum*, and included in a low-supported cluster with *Arsenophonus* symbionts from several whiteflies, bat flies and one ant. The use of a species threshold of 95% ANI (Konstantinidis and Tiedje, 2005) suggested that *A. triatominarum, Candidatus* Arsenophonus nilaparvata, *Arsenophonus* of *Entylia carinata*, ARAF and ARTV belong to the same species, whereas *Arsenophonus nasoniae, Candidatus* Arsenophonus lipopteni and ARAD form three different species (Table S4 and Figure S4). The AAI analysis showed the presence of two clusters, one for ARAD and *A. nasoniae* and the other for the remaining species, except the endosymbiont *A. lipopteni*, with a value ≤80% AAI. The phylogenomics results showed partial congruence with ANI/AAI values, placing *A. triatominarum* as the closest relative of ARAD (Figure 2A). The two endosymbionts formed a sister monophyletic clade to all other *Arsenophonus*, which formed a second monophyletic clade. *A. nasoniae* was placed at the basal position of both clades.

The phylogenetic positions of the two *Wolbachia* were determined with the same analyses. In the 16S rRNA phylogeny, *Wolbachia* WBAD and WBAF formed a well-supported clade inside a major cluster, which contains *Wolbachia* strains from other whiteflies and various insect taxa (Figure S5). In addition, based on phylogenomics, WBAD and WBAF were placed as a basal clade relative to the other *Wolbachia* from the B super group and with a *Wolbachia* from the mosquito *Aedes albopictus* as the closest genome included in the analysis (Figure 2B). Although included in the B super group, *Wolbachia* from *B. tabaci* was placed in a different cluster, closer to a *Wolbachia* from the fly *Drosophila simulans*, and as a sister group of a clade that includes several *Wolbachia pipientis* strains. Finally, the high nucleotide identity (97.6% ANI) between WBAD and WBAF suggested that these bacteria are strains of the same, recently diverged, species.

### 3.3. Comparative genomics: synteny and functional categories

**Figure 3:**
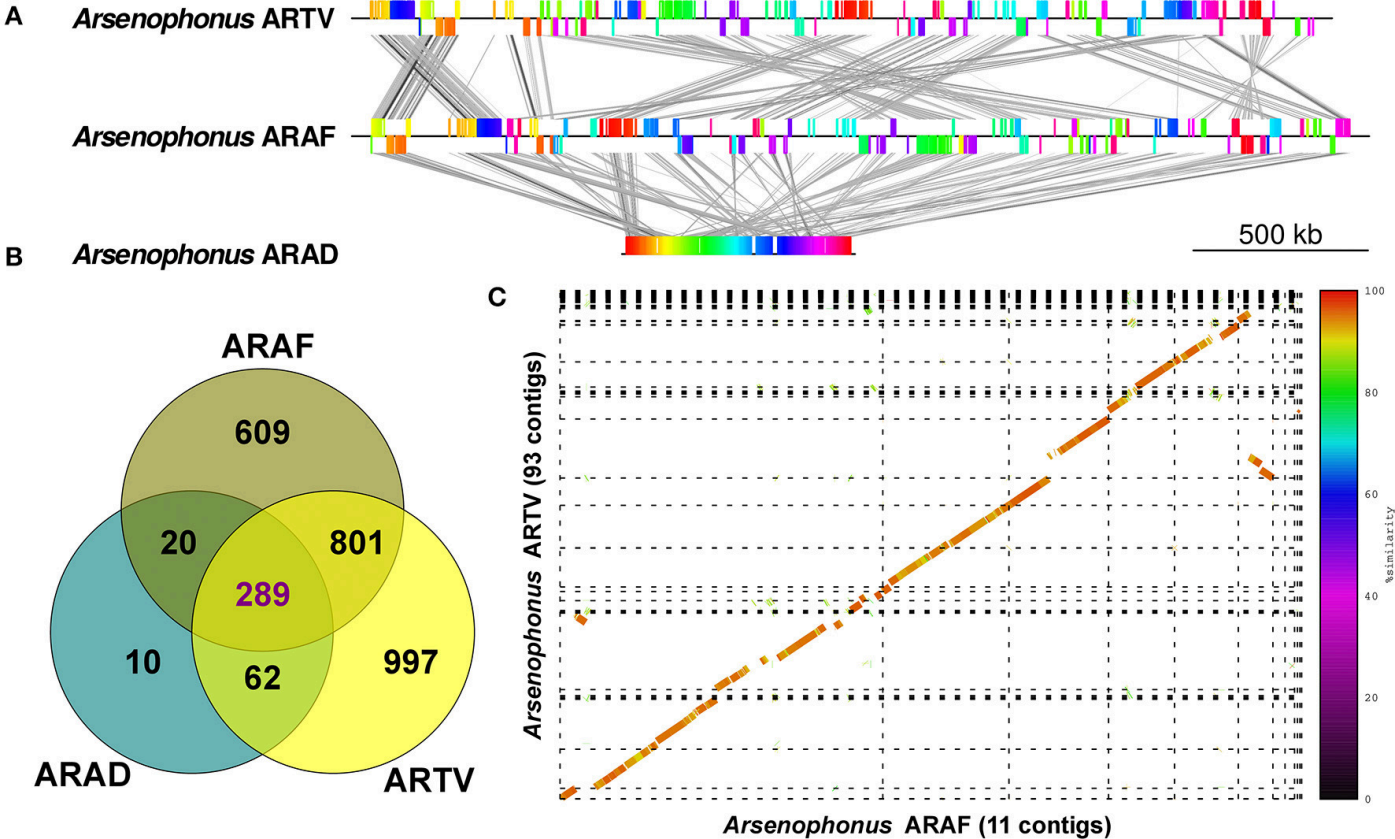
Comparative analyses of the three *Arsenophonus* MAGs. **(A)** Graphic linear representation of the genome rearrangements observed comparing ARTV with ARAF and ARAF with ARAD. Synteny blocks display the same color. ARAF and ARTV contigs were ordered according to ARAD before synteny blocks calculations with Mauve for a better visualization. **(B)** Venn diagram displaying the number of clusters found on each subspace of the *Arsenophonus* pangenome (Table S5). **(C)** Synteny between the draft genomes of ARTV and ARAF. Contig edges are displayed as dashed lines. Contigs of ARTV were ordered with Mauve using ARAF as reference for better visualization.

When the inferred proteomes from *Arsenophonus* ARAD, ARAF, and ARTV were compared (Table S5), a core genome composed of only 289 clusters was obtained (Figure 3B). Interestingly, the core genome included many clusters related to vitamin and cofactor biosynthetic pathways. The small number of shared protein clusters resulted from the reduced proteome of ARAD, which is mainly a subset of the larger ARTV and ARAF proteomes. Only 4 out of the 10 specific ARAD clusters were not hypothetical proteins: pyruvate kinase II (pyruvate kinase I is present in the three proteomes), 3-oxoacyl-[acyl-carrier-protein] synthase (*fabF*), which is involved in fatty acid and biotin biosynthesis, and proteins encoded by *recA* and *nudE*. If only ARAF and ARTV had been compared, the core proteome would have contained more than 1,000 clusters. The number of species-specific clusters of ARTV (997) and ARAF (609) were higher than for ARAD, which is in accordance with their genome sizes. Synteny analysis highlighted the strong genome reduction in ARAD compared to ARAF and ARTV (Figure 3A). This included the loss of macrosynteny (general genome architecture), while maintaining microsynteny (e.g., operons). In contrast, ARTV and ARAD still showed a high level of macrosynteny, although some rearrangements were observed (Figure 3C).

**Figure 4:**
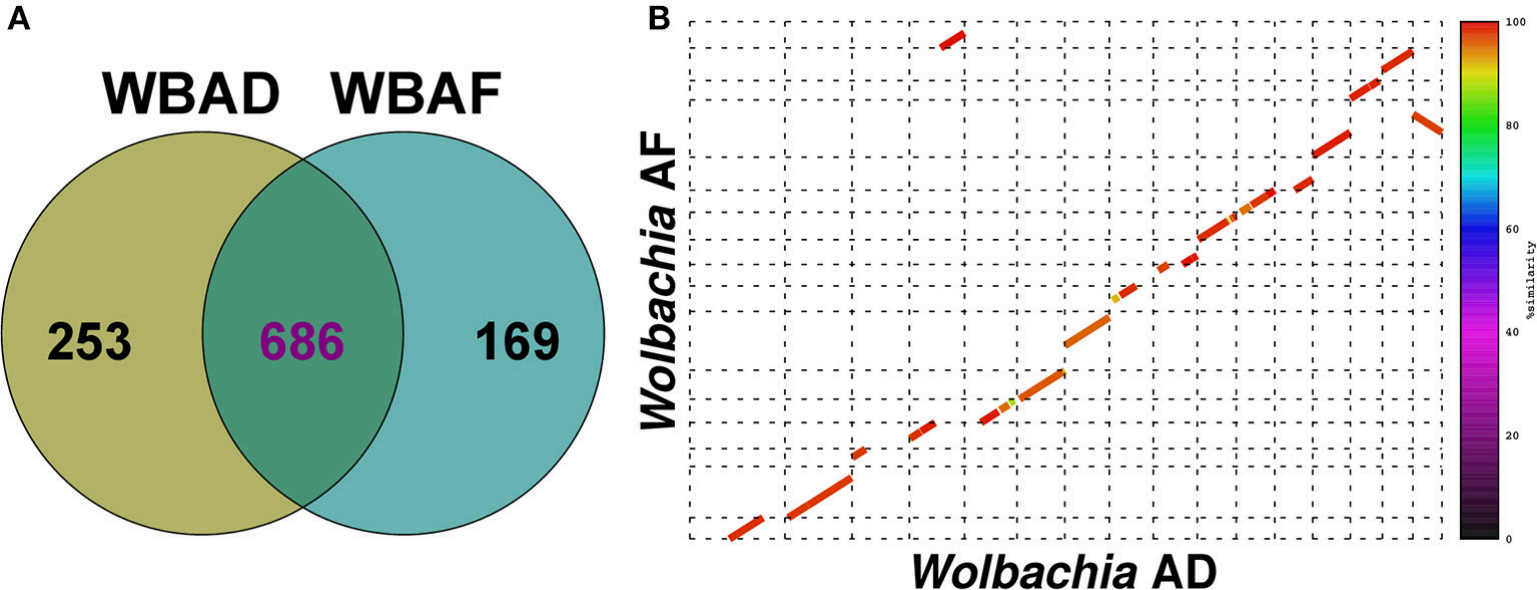
Comparative analyses of the two *Wolbachia* MAGs. **(A)** Venn diagram showing the shared and specific coding gene clusters between WBAD and WBAF (Table S6). **(B)** Synteny between some of the largest contigs from WBAD and WBAF draft genomes. Only contigs larger than 10 kb were used as input, accounting for up to 23% and 25% of the WBAD and WBAF draft genomes. Contigs of WBAF were ordered with Mauve using WBAD as reference for better visualization.

*Wolbachia* WBAD and WBAF showed a core genome of 686 clusters, and 253 and 169 species-specific clusters, respectively (Figure 4A and Table S6). Although some genomic regions (contigs) were strain-specific, it should be noted that the highly fragmented status of these genomes could produce a large number of artificial specific clusters. Comparison of the two *Wolbachia* genomes revealed that some of the largest contigs show the same gene order and a high level of nucleotide identity (Figure 4B), suggesting that at least microsynteny is probably kept. However, due to the draft status of the genomes, it is not clear if macrosynteny is conserved.

**Figure 5:**
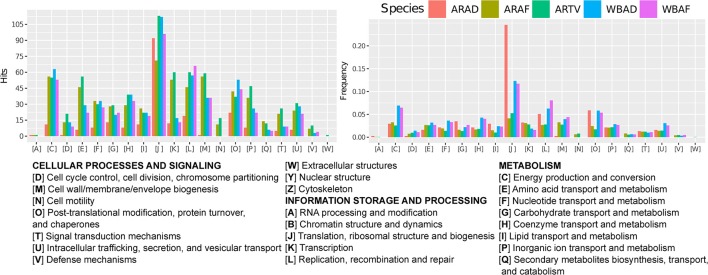
Distribution of the endosymbiont proteomes in functional categories. Bar plots showing the number of hits **(left)** and their relative frequency **(right)** in each functional category (COG) in the proteomes of the three analyzed *Arsenophonus* and the two analyzed *Wolbachia* MAGs.

The proteomes of the five endosymbionts were functionally classified according to COGs. Because of the strong reduction of the gene repertoire in ARAD, this endosymbiont showed a smaller number of hits in each functional category, except J (translation and ribosomal structure and biogenesis), where the number of hits was similar to the other endosymbionts (Figure 5 left). In fact, in ARAD, this category had a relative frequency in the proteome ≥0.20 (Figure 5 right). In addition, category J and the other categories related to information storage and processing (A, K, and L) showed higher relative frequencies in ARAD than in the other two *Arsenophonus* (Figure 5 right). Based on the number of hits and relative frequencies, the loss of the gene repertoire in categories G (carbohydrate metabolism), I (lipid metabolism), and O (post-translational modification and chaperones) was lower in ARAD. As expected, *Wolbachia* proteomes retained the informational categories but also retained COGs related to, among others, environmental response.

### 3.4. Biosynthetic metabolic potential

**Figure 6:**
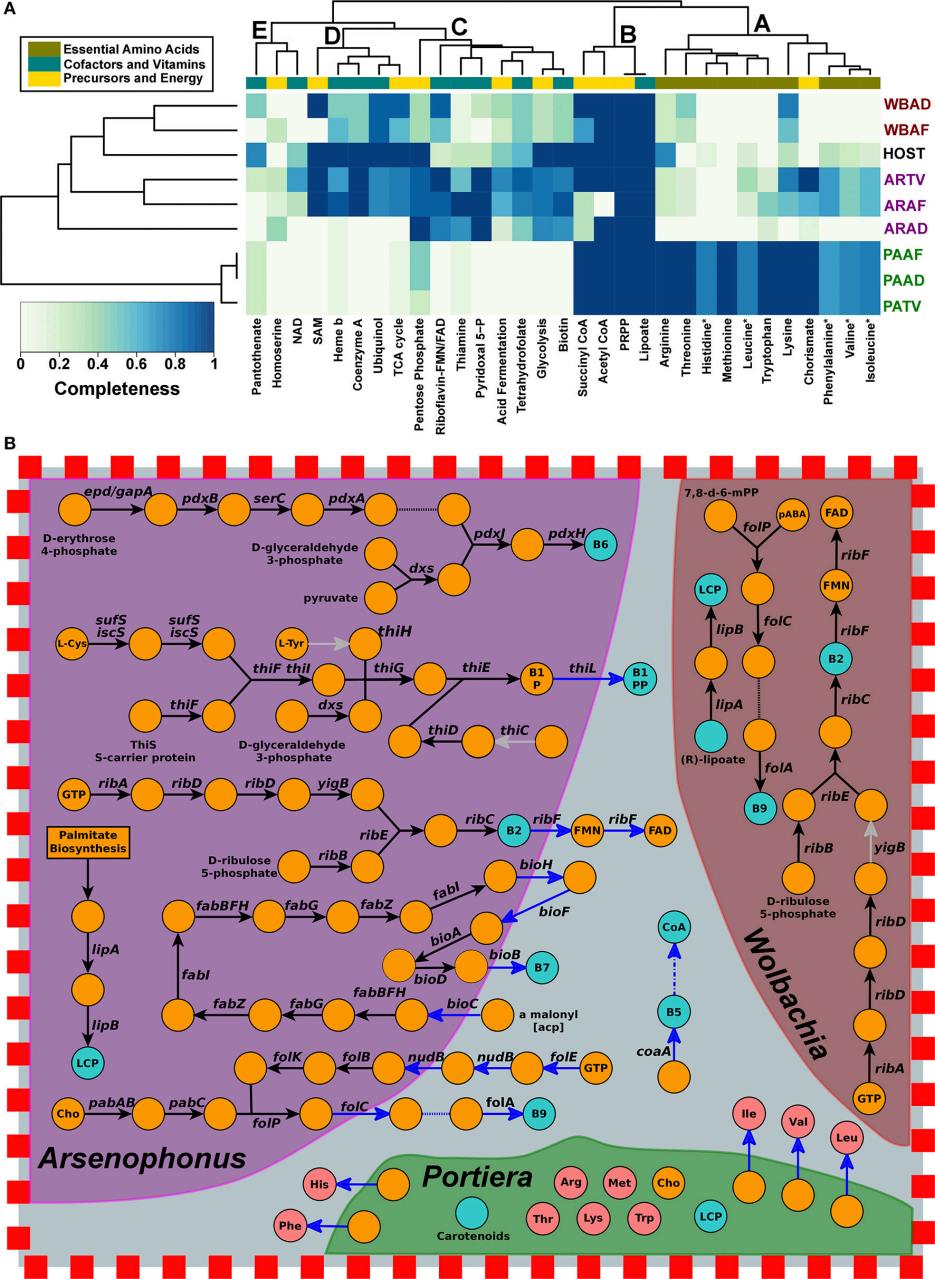
Biosynthetic potential of *Arsenophonus, Wolbachia, Portiera* and whiteflies. **(A)** Heat map and hierarchical clustering showing, on a color scale, the degrees of completeness of several compounds' biosynthetic pathways, including essential amino acids, cofactors and vitamins. *Arsenophonus* (AR), *Wolbachia* (WB) and *Portiera* (PA) from *A. dispersus* (AD), *A. floccissimus* (AF) and *T. vaporariorum* (TV) are highlighted in purple, red, and green, respectively. *B. tabaci* genome was used as a general host representative. **(B)** Schematic representation of some vitamin/cofactor and precursor metabolic pathways occurring in the bacteriocytes of *A. dispersus*. Orange, blue, and pink circles represent intermediate metabolites, vitamins/cofactors and essential amino acids, respectively. Black arrows denote enzymes present in the corresponding endosymbiont, blue arrows enzymes present in the host, light gray arrows pseudo/absent genes, dashed arrows simplified pathways, and dashed lines spontaneous reactions. Abbreviations: lipoyl carrier protein (LCP), chorismate (Cho), acyl carrier protein (acp), 4-aminobenzoate (pABA), (7,8-dihydropterin-6-yl)methyl diphosphate (7,8-d-6-mPP), diphosphate (PP).

An integrated analysis of the metabolism of the bacteriocytes of the three whitefly species was performed to predict the potential biosynthetic capabilities of the harbored *Arsenophonus, Wolbachia* and *Portiera* (Santos-Garcia et al., 2015). That of the insect host was inferred using the *B. tabaci* genome (GCA_001854935.1). Amino acid biosynthesis in the three whiteflies was mainly conducted by their *Portiera* strains [(*P. aleyrodidarum* from *A. dispersus* (PAAD), *A. floccissimus* (PAAF) and *T. vaporariorum* (PATV)], which maintained the ability to produce 4 (PATV) or 5 (PAAF and PAAD) of the 10 essential amino acids, and required some complementation from the host for the synthesis of the others (Figure 6A, cluster A). While some cofactors/vitamins and precursors were produced by almost all of the endosymbionts and the host (cluster B), others were mainly produced by some *Arsenophonus* (cluster C), both ARAF and ARTV, both *Wolbachia* and the host (cluster D), or, apparently, none of them (cluster E). In general, both *Wolbachia* presented lower biosynthetic potential than that of the most reduced *Arsenophonus*, ARAD. In addition, while both *Wolbachia* still presented all of the electron transport chain, ARAF and ARTV lost ubiquinol oxidase and ARAD only maintained cytochrome-c oxidase but lost ATP synthase (not shown).

Although the potential for essential amino acid biosynthesis of *Arsenophonus* and *Wolbachia* was limited (Figure 6A), ARAF and ARTV still retained some genes involved in the synthesis of amino acids or their precursors, such as lysine (its precursor, meso-diaminopimelate is also involved in peptidoglycan biosynthesis) and chorismate (the precursor of phenylalanine). In ARTV, the complete pathway, encoded by seven genes, from D-erythrose 4-phosphate and D-ribulose-5-phosphate to chorismate was functional (*aroF, aroB, aroQ, aroE, aroL, aroA*, and *aroC*). This, in combination with the presence of *tyrA, tyrB*, and an additional monofunctional chorismate mutase gene, suggests ARTV's ability to produce tyrosine and its potential collaboration with *Portiera*, or the host, for the production of phenylalanine. The ability to synthesize amino acids was completely lost in ARAD, suggesting that this not the reason why the lineage of ARAD evolved a close association with *A. dispersus*.

The contribution of the different partners to the synthesis of vitamins and cofactors was heterogeneous. ARTV and ARAF, the two *Arsenophonus* with larger genomes, maintained higher capabilities. Although the possibility of missed genes in the process of read recovery and assembly cannot be ruled out, ARTV and ARAF seem to have lost several genes required for some of the pathways. Despite the reductive evolutionary process in ARAD that has drastically reduced its potential, some capabilities are still retained, suggesting that they might be important in the symbiotic relationship (Figures 6A,B). ARAD, but also ARAF and ARTV, encode the complete biosynthesis pathway for pyridoxal 5′-phosphate (vitamin B6) and riboflavin (vitamin B2). However, while ARAF and ARTV are predicted to be able to produce flavin mononucleotide (FMN) and flavin adenine dinucleotide (FAD) because they harbor a functional *ribF* gene, it is pseudogenized in ARAD. Since this gene is present in all whitefly hosts, both flavin cofactors may be produced in *A. dispersus* through complementation between ARAD and its host (Figure 6B, Table S2). In addition, considering functions that can be complemented by the host, ARAD is predicted to be able to synthesize thiamine (vitamin B1) and folate (vitamin B9, from imported chorismate) (Figure 6B). Although ARAD has lost *thiH*, some host-encoded proteins could potentially replace its function (Table S2). ARAF and ARTV also require complementation by their host to produce folate. ARTV has lost most of the thiamine-production pathway, and the missing steps are not complemented by its host. In addition, *B. tabaci* seems to be able to produce biotin using some horizontally transferred genes (*bioA, bioB*, and *bioD*) (Luan et al., 2015), a protein capable of replacing the BioC function, and the insect's own fatty acid biosynthesis pathway. Genes with similar function to *bioABCDFH* genes were found to be present in *T. vaporariorum* genomic reads, suggesting that this whitefly is also capable of producing biotin by itself. Because only genes similar to *bioBCFH* were identified in the genomic reads from both *Aleurodicus* species (Table S2), the presence of *bioA* and *bioD* in ARAD and ARAF is required for the cooperative synthesis of biotin. However, ARAD, unlike ARAF, has only retained the two missing genes in the genomes of both *Aleurodicus*.

In ARAF, the gene *yigB* which is part of the riboflavin pathway, was pseudogenized. This gene encodes a Haloacid Dehalogenase (HAD) phosphatase that belongs to a superfamily of enzymes. Therefore, the activity of the protein encoded by this gene can likely be substituted by related genes in the endosymbiont or host genomes (Manzano-Marín et al., 2015). Finally, all *Arsenophonus* conserved the glycolysis and pentose phosphate pathways to produce the precursors required for riboflavin, thiamine, and pyridoxal. Indeed, in ARAD, the enzyme encoded by *gatA* from the glycolysis pathway seems to have replaced the *epd* product in the pyridoxal pathway. The combination of the *ilvC*-encoded enzyme in *Portiera* (Price and Wilson, 2014) and the *panBC* genes that were horizontally transferred to *B. tabaci* allows this whitefly to produce its own pantothenate (vitamin B5) and coenzyme A. However, *panBC* genes are not likely to be present in the genomes of *A dispersus, A. floccissimus*, or *T. vaporariorum*, where only bacterial hits were recovered, showing no similarity to the ones present in the *B. tabaci* genome. This suggests that these whiteflies are not able to produce pantothenate and they acquire it from the diet or other sources (Figure 6B and Table S2). However, failure of horizontally transferred gene detection could be an artifact of the methods used, since they were unable to discover new horizontally transferred genes in the screened whiteflies.

The third endosymbiont of *A. dispersus* and *A. floccissimus, Wolbachia* WBAD and WBAF, may also contribute to the vitamin/cofactor synthesis of the system. Similar to *Arsenophonus* ARAD and ARAF, they encode the complete pathway for riboflavin–FMN–FAD synthesis and might be able to produce folate from some intermediate precursors (Figure 6B). In addition, WBAD and WBAF are able to activate lipoate. However, as indicated above, we cannot rule out the presence of other biosynthetic pathways that were lost in the WBAD and WBAF genomes due to the large fragmentation of the assemblages. The same could be happening with respect to incomplete pathways, where gaps may simply reflect genes that were missed during the assembly process.

## 4. Discussion

Some bacterial lineages show predominantly facultative symbiotic lifestyles with insects. In such cases, they can inhabit the cytoplasm of bacteriocytes and coexist with the primary endosymbiont. These bacterial lineages usually have large gene repertoires and genomes, allowing them to be autonomous and capable of infecting individuals from very different taxa. Examples of such clades are the genera *Arsenophonus, Sodalis*, and *Serratia* (Nováková et al., 2009; Manzano-Marín et al., 2017; Santos-Garcia et al., 2017). Because their presence may have fitness costs for the host, they do not become fixed in the insect lineage except if their nutritional contribution (or other kind of benefit) is important and is required for multiple generations. Under such a scenario, a facultative endosymbiont may evolve toward a more intimate association, becoming a co-primary symbiont (Lamelas et al., 2011). Although there are several features associated with this new lifestyle, the most relevant is a decrease in genome size. In the lineage *Arsenophonus*–*Riesia*, the sizes of several genomes have been reported, ranging from 0.58 Mb for *Candidatus* Riesia pediculicola (Kirkness et al., 2010) and 0.84 Mb for *A. lipopteni* (Nováková et al., 2016) to 3.86 Mb for *A. triatominarum*, but with several genomes of intermediate size (Šochová et al., 2017). Similarly, *Sodalis* endosymbionts display a large range of genome sizes, from 0.35 Mb for *Candidatus* Mikella endobia (Husnik and McCutcheon, 2016) to circa 4.5 Mb for *Sodalis glossinidius* or *Candidatus* Sodalis pierantonius, and with several genome sizes in the range of 1–2 Mb (Santos-Garcia et al., 2017). This range has also been observed in *Serratia symbiotica*, with genome sizes from 0.65 to 3.86 Mb (Manzano-Marín et al., 2016). To the best of our knowledge, *Arsenophonus* ARAD is among the smallest genomes in its genus, between *R. pediculicola* and *A. lipopteni* (Šochová et al., 2017). ARAD is still undergoing a genome reduction process that could end up ≤0.4 Mb if the current 54% coding density is considered. The ARAD gene repertoire is clearly a subset of *Arsenophonus* from other whiteflies'. It is totally dependent on the host environment and, as we discuss below, putatively supplies/complements its host with cofactors/vitamins that are not produced by *Portiera* and/or the host. Thus, ARAD can be considered a co-primary endosymbiont. On the other hand, ARAF and ARTV are still at an intermediate stage of the genome-reduction process (e.g., high number of pseudogenes, inactivation of mobile genetic elements, and virulence/secretion factors), but they are already showing dependence on the host environment. Therefore, they can be considered relatively recent obligate endosymbionts with the potential to establish a co-primary symbiotic relationship with *Portiera* and their host (Latorre and Manzano-Marín, 2016).

Based on phylogenetic and molecular methods, Thao and Baumann (2004) indicated that whiteflies have experienced multiple *Arsenophonus* infections. Nováková et al. (2009) phylogenetic analysis placed these *Arsenophonus* from whiteflies in five, probably six, lineages in a broader phylogeny that includes several *Arsenophonus* from different hosts. Despite some topological differences produced by low-supported clades, our results are in accordance with both studies. With respect to the *Arsenophonus* 16S rRNA phylogenetic relationships, the long branches of the *A. dispersus*/*A. dugesii* lineage, which includes ARAD, suggest a more ancient infection compared to *Arsenophonus* from other insects, including whiteflies (with the exception of *B. centroamericana*). In addition, it should be noted that a second *Arsenophonus* from *A. dugesii* presented a shorter branch and was closer to *B. centroamericana*. This suggests a potential endosymbiont replacement, closely related to the one in *B. centroamericana*, in this *Aleurodicus* species. The ARTV/ARAF cluster contains several whitefly species and some of them may have been infected by the same, or closely related, *Arsenophonus* strains. In the case of ARAF, the long branch is an artifact of the incompletely recovered copy of the 16S rRNA gene, and not the result of a long-term association with its host. Nevertheless, our phylogenomic and ANI/AAI analyses strongly support ARAD's belonging to a different lineage from the other two whiteflies' *Arsenophonus*, ARAF and ARTV. Two scenarios can explain the fact that ARAD and ARAF, despite their host belonging to the same genus, are from two different lineages. In the first, the ancestor of ARAD infected the lineage of *A. dispersus* after its divergence from *A. floccissimus* and, later, started its shift to a primary symbiosis and its associated genome-reduction process. In this case, the process could have taken place for as long as 18 million years (My), the estimated divergence time between these two whitefly species (Santos-Garcia et al., 2015). An alternative scenario is that this association began prior to the divergence of both *Aleurodicus* species, but later, the ARAD-type symbiont was replaced in the *A. floccissimus* lineage by another *Arsenophonus* lineage (ARAF). Although both scenarios are possible, the large number of pseudogenes in the ARAD genome suggests that the initiation of the process was relatively recent, as suggested by the first scenario. For example, *Riesia*, which also shows a strong genome reduction, started its symbiosis with *Pediculus* lice around 13–25 My ago (Mya) (Kirkness et al., 2010). Still, the second scenario cannot be excluded as symbionts, independent of their symbiotic status, can be replaced by other bacteria of the same, or different genera, as long as the newcomers have similar biosynthetic capabilities. Indeed, repeated symbiont replacements have been documented in many insect lineages, including other sternorrhynchans such as aphids and mealybugs (Husnik and McCutcheon, 2016; Manzano-Marín et al., 2017; Meseguer et al., 2017; Russell et al., 2017; Sudakaran et al., 2017). Regardless of whether ARAD was already present in the *Aleurodicus* ancestor, and taking into account that this genus diverged from the *Trialeurodes* (Aleyrodinae) ancestor more than 100 Mya (Shcherbakov, 2000; Drohojowska and Szwedo, 2015), it is clear that both ARAF and ARTV originated from independent recent infections by the same, or closely related, *Arsenophonus* strains. Previous works have demonstrated that horizontal transmission of endosymbionts between whiteflies can be plant and/or parasitoid mediated; however, all reported cases have involved endosymbionts that are not restricted in the bacteriocyte (Chiel et al., 2009; Caspi-Fluger et al., 2012; Ahmed et al., 2015). The fact that all reported *Arsenophonus* are bacteriocyte-restricted makes this hypothesis less plausible (Gottlieb et al., 2008; Skaljac et al., 2013; Marubayashi et al., 2014; Pandey and Rajagopal, 2015) and gives more support to the idea that an opportunistic *Arsenophonus*, for example one that was gut-associated or from other insects but with the ability to invade new hosts, was the source of those infections. Later on, during the establishment of this *Arsenophonus* in the new host's environment, it could derive into an obligate endosymbiont, as in the case of ARAF and ARTV.

Nutritionally poor diets, such as phloem sap or blood, cannot provide insects with all of their dietary requirements, including several vitamins. For example, the essentiality of dietary riboflavin was demonstrated in aposymbiotic aphids, but not in aphids harboring their primary endosymbiont *Buchnera aphidicola*, which is able to produce riboflavin (Nakabachi and Ishikawa, 1999). Moreover, *B. aphidicola* of aphids from the subfamily Lachninae had already lost the genes encoding the pathway in the Lachninae ancestor. This led to the establishment in this group of a new association with an additional bacterium, capable of producing riboflavin. In most lineages, this is the co-primary *S. symbiotica*, a facultative endosymbiont in other aphid lineages (Manzano-Marín and Latorre, 2014). The analyses of the co-symbiosis in species of Lachninae revealed a complex system with *S. symbiotica* endosymbionts harboring a very small genome (in *Tuberolachnus salignus*) or intermediate genome sizes (in some species of the genus *Cinara*), or being replaced by other endosymbionts belonging to different genera, such as *Sodalis, Erwinia*, or *Hamiltonella*, among others (Manzano-Marín et al., 2016, 2017; Meseguer et al., 2017). The potentially essential role of riboflavin biosynthesis in establishing symbiotic relationships has also been demonstrated in several blood-feeding invertebrates harboring different endosymbionts, such as the leech *Haementeria officinalis* and its *Candidatus* Providencia siddallii endosymbiont (Manzano-Marín et al., 2015), the *Wolbachia* associated with the bedbug *Cimex lectularius* (Moriyama et al., 2015), or the *Coxiella*-like bacteria found in several ticks (Gottlieb et al., 2015). Indeed, the *Arsenophonus*–*Riesia* lineage includes several examples of intimate host–symbiont associations with the production of B vitamins as a pivotal role, but also several facultative symbionts of this lineage have the potential to produce vitamins and cofactors. For example, the louse fly *Melophagus ovinus* is a hematophagous dipteran that relies on *Candidatus* Arsenophonus melophagi, its primary endosymbiont, to acquire B vitamins that are not present in its diet, i.e, riboflavin, pyridoxine, and biotin (Nováková et al., 2015). *A. lipopteni* is the primary endosymbiont of another hematophagous louse fly (*Lipoptena cervi*), and similar to *A. melophagi*, it has the potential to produce riboflavin, pyridoxine, and biotin (Nováková et al., 2016). In the human louse *Pediculus humanus*, another hematophagous insect, *R. pediculicola* is in charge of supplying its host with several B vitamins including pantothenate, riboflavin, and biotin (Kirkness et al., 2010). *A. nasoniae* from the parasitic wasp *Nasonia vitripennis* has broad metabolic potential, including the ability to produce riboflavin, biotin, folate, thiamine, and pyridoxine (Darby et al., 2010). The brown planthopper *Nilaparvata lugens* feeds exclusively on rice phloem and has a yeast-like primary endosymbiont that produces essentials amino acids, and helps its host with nitrogen recycling but also steroids synthesis. However, *N. lugens* always has *Arsenophonus* or *Wolbachia* as secondary endosymbionts. Again, the *Arsenophonus* from *N. lugens* has broad metabolic potential and is able to produce riboflavin, biotin, folate, thiamine, and pyridoxine (Xue et al., 2014). Similar biosynthetic potential was found in the *Arsenophonus* from the treehopper *Entylia carinata*, another phloem-feeding insect, although in this case *Arsenophonus* is not fixed in the population (Mao et al., 2017). Interestingly, *Arsenophonus* seems to be the most common secondary endosymbiont found in whiteflies, always sharing the bacteriocytes with *Portiera* and in most cases being fixed (or close to it) in the tested populations (Thao and Baumann, 2004; Gottlieb et al., 2008; Skaljac et al., 2013; Cass et al., 2014; Kapantaidaki et al., 2014; Marubayashi et al., 2014; Pandey and Rajagopal, 2015; Santos-Garcia et al., 2015). Therefore, taking into account the metabolic potential of other *Arsenophonus* infecting different hosts, we hypothesize that the ability to synthesize riboflavin and other vitamins is probably the main reason for the presence of *Arsenophonus* endosymbionts in whiteflies and for the evolution of ARAD toward a co-primary symbiont in *A. dispersus*. In such a scenario, the continuous acquisitions/replacements of *Arsenophonus* in whiteflies could maintain, at the population level, the availability of such vitamins when the concurrent secondary endosymbiont is no longer able to produce them, or when a newcomer with greater metabolic potential and the ability to establish symbiotic relationships arrives.

Although thiamine, pantothenate, coenzyme A, niacin, pyridoxal and biotin can be found in the phloem (free or bound to transporter proteins) (Zimmermann and Milburn, 1975), their concentrations might not be sufficient for many phloem-feeding species. This could explain why the ability to produce some of these compounds has been retained in bacterial endosymbionts whereas it has been lost in others. However, the production of one or several B vitamins (thiamine, riboflavin, pyridoxal, folate, and biotin) and *de novo* lipoate by whiteflies' *Arsenophonus* seems to be the main contribution of these endosymbionts to their hosts, allowing them to feed on the limited diet present in the phloem (Dale et al., 2006; Nováková et al., 2015, 2016; Mao et al., 2017). Regarding biotin biosynthesis, the bacterial origin of *bioAB* and *bioD* was reported by Luan et al. (2015) and Ankrah et al. (2017). We show here that not all whiteflies seem to have the horizontally transferred *bioAD* genes, although the rest of the *bio* operon homologs, including *bioB*, are present. This suggests that some whiteflies acquired, by several horizontal gene-transfer events, the ability to produce biotin. This is expected to produce a gradual loss of the biotin pathway in secondary endosymbionts of the different whitefly lineages, at least from the Aleyrodinae subfamily, as this group harbors the full *bio* operon. Indeed, *Cardinium* infecting *B. tabaci* has recently lost its ability to produce biotin, raising the possibility that biotin is provided by the co-present *Hamiltonella* endosymbiont or by the host's metabolic potential (Santos-Garcia et al., 2014b; Rao et al., 2015). As an alternative, this can also lead to intricate complementation patterns as shown in the *Arsenophonus* from *Aleurodicus*. It is important to notice that supplementation of vitamins was the role proposed for *Hamiltonella* in *B. tabaci* (Rao et al., 2015) and the results reported here support a similar function for *Arsenophonus*. These two endosymbionts are generally never found together in the same bacteriocyte (Gottlieb et al., 2008; Skaljac et al., 2013; Marubayashi et al., 2014). It could be that *Hamiltonella* and *Arsenophonus* compete for the same host resources to produce the same compounds, undermining their unique benefit to the host if harbored together due to the associated cost of maintaining different endosymbionts at high loads (Ferrari and Vavre, 2011). Interestingly, Skaljac et al. (2010) reported that *Hamiltonella* and *Arsenophonus* are, apparently, found together in *T. vaporariorum* bacteriocytes, and present the same sub-cellular localization. However, a worldwide *T. vaporariorum* population study, conducted by Kapantaidaki et al. (2014), concluded that this whitefly is a single species with low levels of intraspecific variation. In addition, *Arsenophonus* was almost fixed in all populations, no presence of *Hamiltonella* was detected, and only sporadic infections of *Wolbachia, Cardinium* and *Rickettsia* were reported (a few individuals per population) (Kapantaidaki et al., 2014). Similar results were shown by Marubayashi et al. (2014) in Brazilian *T. vaporariorum* populations, where *Arsenophonus* was fixed in the population and no *Hamiltonella* co-infection was recorded. Indeed, our *T. vaporariorum* sequencing results support these latter studies, with few reads classified as *Hamiltonella* (216) and these possibly being misclassifications, and *Wolbachia* previously detected by PCR (Santos-Garcia et al., 2015) but with few reads assigned to it.

Finally, and in contrast to *Arsenophonus, Wolbachia* from *Aleurodicus* species only produces riboflavin *de novo* and, probably, folate and lipoate from some intermediate metabolites. It has been shown that the bedbug *C. lectularius* requires the riboflavin produced by *Wolbachia* for its proper development. Indeed, the riboflavin pathway is conserved among *Wolbachia* species infecting different invertebrates, suggesting that it may provide a fitness benefit to the infected host (Moriyama et al., 2015). However, it should be noted that while *Hamiltonella* and *Arsenophonus* are restricted to the bacteriocytes, *Wolbachia* is also found in other tissues/cells (Gottlieb et al., 2008; Skaljac et al., 2010, 2013; Bing et al., 2014; Marubayashi et al., 2014). While the first pattern is more characteristic of hemipteran mutualistic endosymbiosis, the second can be found in endosymbionts with a wide range of symbiotic interactions, including parasitism. In addition, these mutualistic endosymbionts are usually fixed, or close to fixed, in different insect populations, as in the case of *Arsenophonus* in *T. vaporariorum* or *Trialeurodes abutiloneus* (Cass et al., 2014; Kapantaidaki et al., 2014), or *Hamiltonella* in *B. tabaci* MEAM1 and MED-Q1 species (Zchori-Fein et al., 2014). However, in most reported cases of whiteflies harboring *Wolbachia*, the individual insects usually also harbor *Arsenophonus* or *Hamiltonella* (Skaljac et al., 2010, 2013; Kapantaidaki et al., 2014; Marubayashi et al., 2014; Zchori-Fein et al., 2014). Nevertheless, special attention should be given to some Asian species of *B. tabaci* and to the ash whitefly *Siphoninus phillyreae*. While *Wolbachia* from *B. tabaci* AsiaII-1 and AsiaII-3 species seem to be fixed at the population level, a mid-to-high prevalence of *Arsenophonus* can also be observed (Bing et al., 2013a). In addition, although *Wolbachia* from *B. tabaci* China1 is fixed in the population, another bacteriocyte-restricted endosymbiont, *Candidatus* Hemipteriphilus asiaticus, is found in this species (Bing et al., 2013a,b). Whether *Wolbachia* contributes to the host diet on these *B. tabaci* species when no other bacteriocyte-restricted secondary endosymbiont is present, or if whether it is the sole source of riboflavin, warrants further investigation. In *S. phillyreae, Wolbachia* seems to be confined to the bacteriocyte, but it is far from being fixed in the tested populations, undermining the importance of this endosymbiont for whitefly nutrition (Skaljac et al., 2013). Finally, the other few cases in which *Wolbachia* was found to be the unique secondary endosymbiont should be handled with caution, as they may simply result from failure of general PCR primers to amplify new symbiont species/strains (Augustinos et al., 2011), or the presence of previously unknown endosymbionts such as *Hemipteriphilus*. In summary, tissue tropism, population infection levels, and genome information suggest that unlike *Hamiltonella* and *Arsenophonus, Wolbachia* is not a mutualistic endosymbiont, at least at the metabolic level, for whiteflies.

In conclusion, the loss of genes encoding the enzymes for the synthesis of vitamins in the ancestral *Portiera*, many millions of years ago, likely generated the requirement of a co-symbiont in whiteflies. *Arsenophonus* species seem to be the most common co-symbiont. In the lineage of *A. dispersus*, the harbored *Arsenophonus* lineage presents a highly reduced genome content. This *Arsenophonus* lineage is, or is in the process of becoming, a co-primary symbiont which putatively supplies/complements its host with cofactors/vitamins that are not produced by its co-partner *Portiera*.

## Data availability statement

The *Arsenophonus* (ERZ502704-6) and *Wolbachia* (ERZ502707-8) annotated genomes and the whole genome shotgun libraries (ERR2532344-45 and ERR2534068-71) analyzed for this study have been deposited in the European Nucleotide Archive (ENA) under project number PRJEB26014. The generated Pathway Tools databases and the blastx results of the genomic reads similar to *Bemisia tabaci* key cofactors/vitamins metabolic genes can be found at https://doi.org/10.6084/m9.figshare.6142307.v1.

## Author contributions

DS-G and FS conceived the study. DS-G and FS performed bioinformatics analysis. KJ performed the phylogenetic analysis. All authors analyzed and discussed the data. DS-G and FS drafted the manuscript with input from AL, AM, EZ-F, SF, and SM. All authors participated in the revision of the manuscript.

### Conflict of interest statement

The authors declare that the research was conducted in the absence of any commercial or financial relationships that could be construed as a potential conflict of interest.
