# Supplementary material for: To B or Not to B: Comparative Genomics Suggests Arsenophonus as a Source of B Vitamins in Whiteflies
Source: Front Microbiol. 2018 Sep 25;9:2254. doi: 10.3389/fmicb.2018.02254 (PMC6167482; doi:10.3389/fmicb.2018.02254)
Supplement: Supplementary file 7 [file Data_Sheet_1.PDF]

Supplementary Figures:

To B or not to B: *Arsenophonus* as a source of B-vitamins in whiteflies.

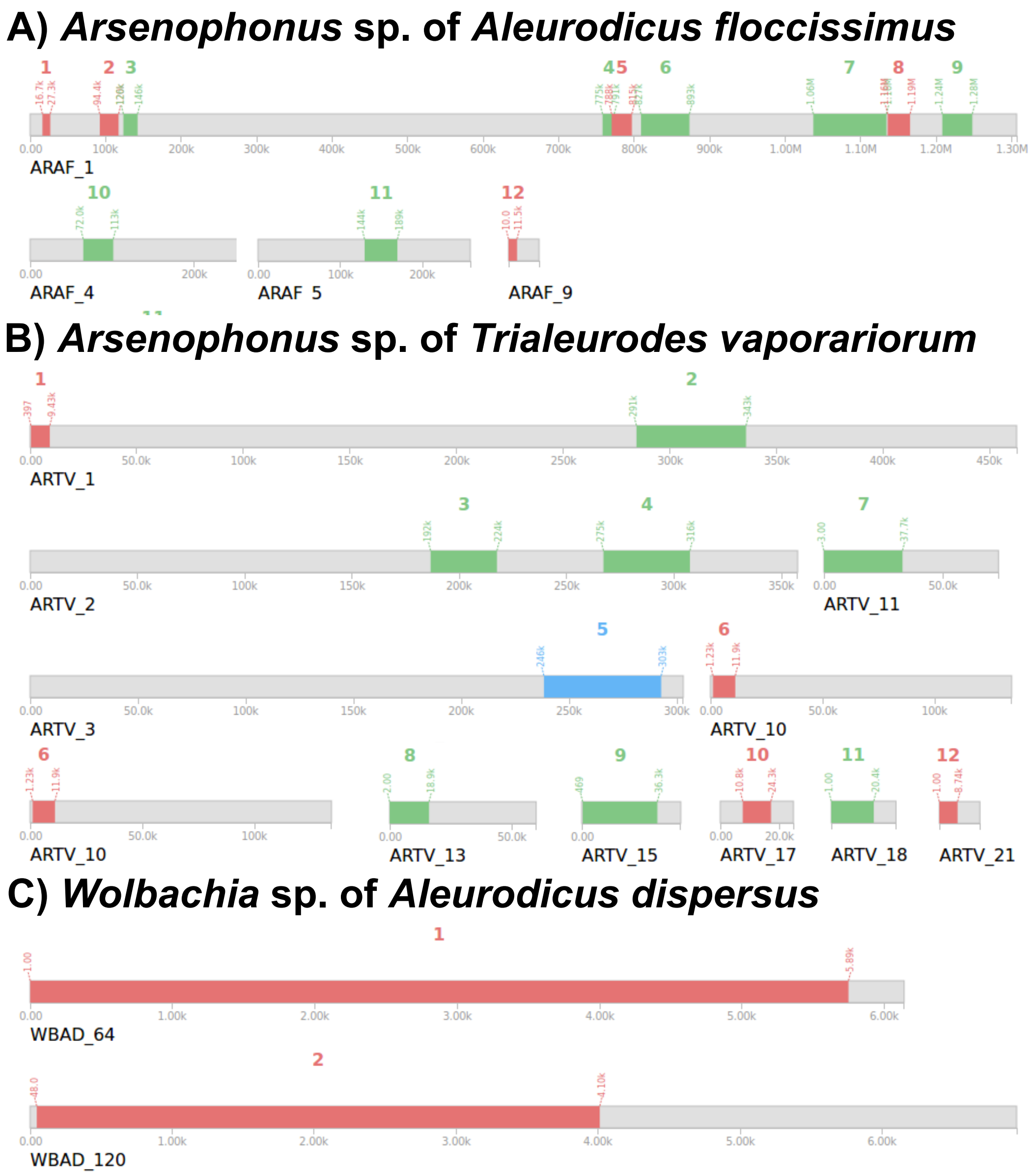

Figure S1. Prophages regions detected in *Arsenophonus* **(A)** ARAF, **(B)** ARTV and **(C)** *Wolbachia* WBAD. Red bars represents incomplete prohages, green bars complete prophages and blue bars questionable complete prophages.

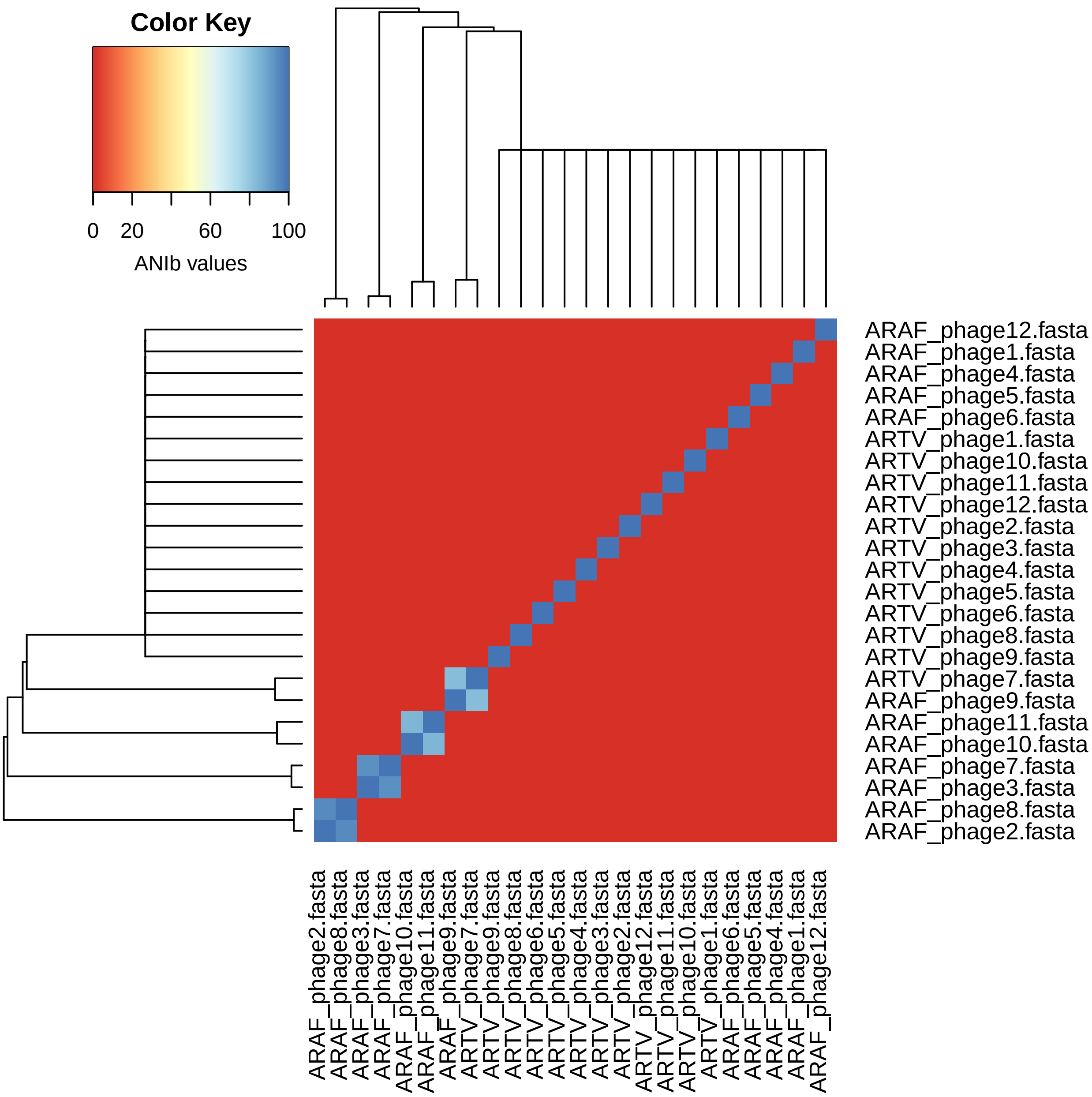

Figure S2. Hierarchical clustering of pairwise Average Nucleotide Identity (ANI) between the *Arsenophonus* ARAF and ARTV detected prophages.

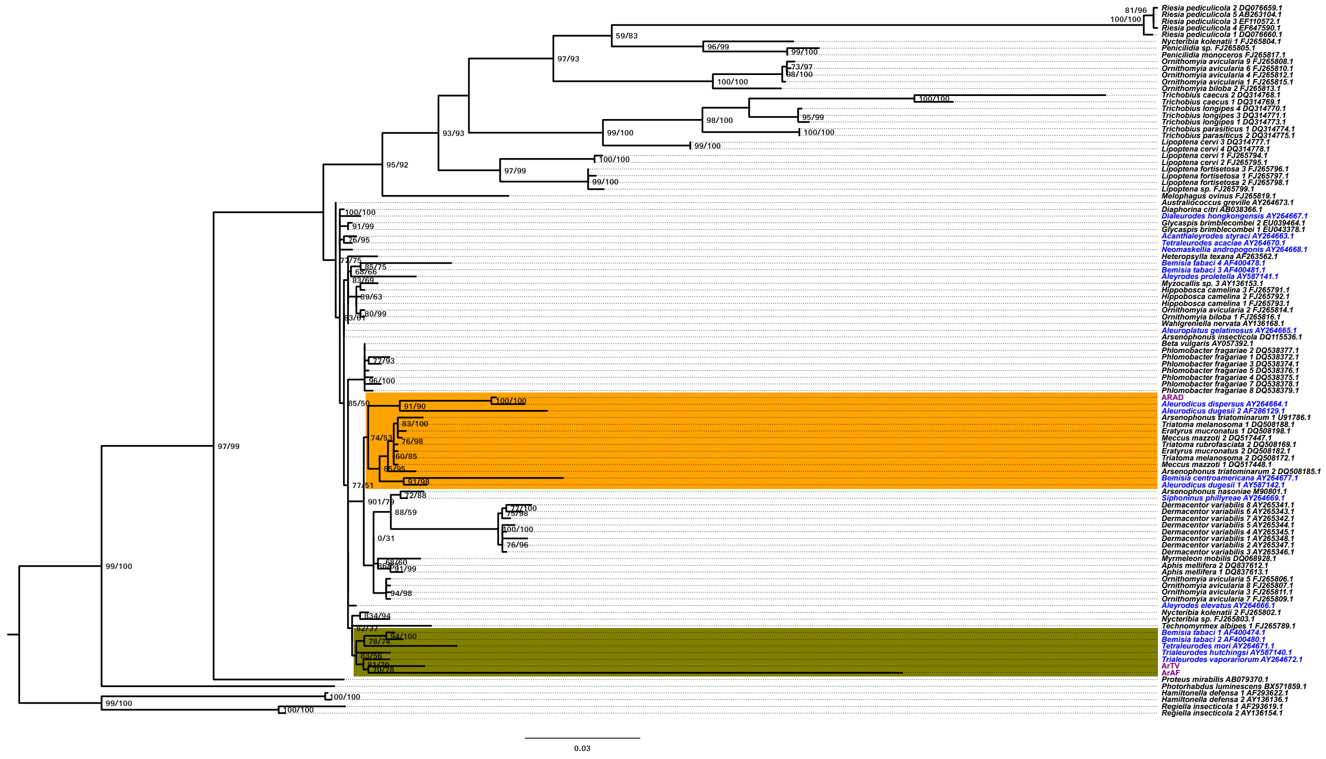

Figure S3. Maximum likelihood tree obtained for several *16S rRNA* genes from *Arsenophonus* infecting different hosts. The tree was inferred using a SYM+R3 substitution model and 5000 ultrafast bootstraps (right node labels) and 5000 SH-aLRT (left node labels). *Arsenophonus* from *Aleurodicus dispersus* (ARAD), *A. floccissimus* (ARAF) and *Trialeurodes vaporariorum* (ARTV) are highlighted in purple. *Arsenophonus* from different whiteflies are highlighted in blue. The orange box highlights sequences grouping to the ARAD cluster while the green box highlight the ones grouping to the ARAF and ARTV cluster.

.

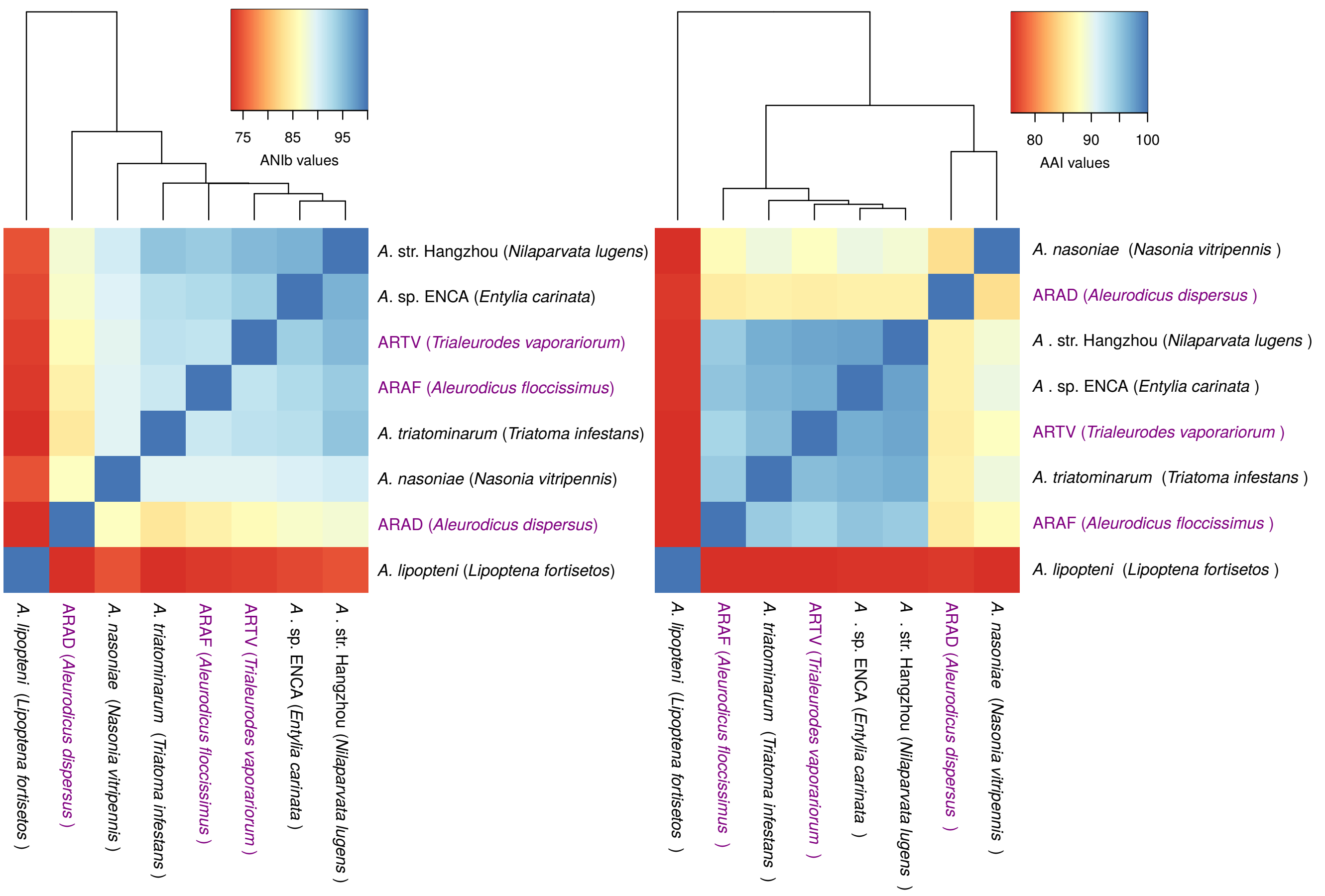

Figure S4. Hierarchical clustering of pairwise Average Nucleotide Identity (ANI, left) and Average Amino Acid Identity (AAI, right) of several *Arsenophonus* genomes. *Arsenophonus* from *Aleurodicus dispersus* (ARAD), *A. floccissimus* (ARAF) and *Trialeurodes vaporariorum* (ARTV) are highlighted in purple. Names for the eukaryotic hosts are shown after the strain names inside parentheses.

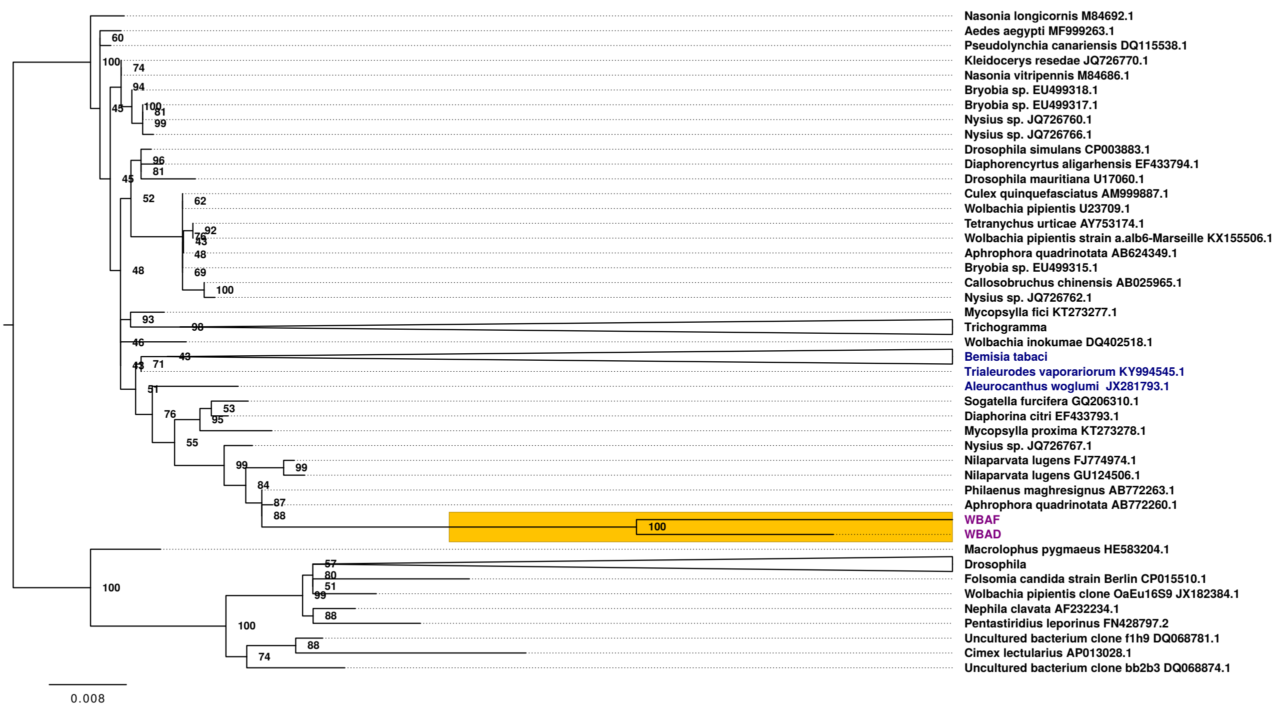

Figure S5. Midpoint rooted maximum likelihood tree obtained for several *16S rRNA* genes from *Wolbachia* infecting different hosts. The tree was inferred using a TN+F+R2 substitution model and 5000 ultrafast bootstraps (node labels). *Wolbachia* from *Aleurodicus dispersus* (WBAD) and *A. floccissicums* (WBAF) are highlighted in purple. *Wolbachia* from different whiteflies are highlighted in blue.
